# From 0D to 2D: N-doped carbon nanosheets for detection of alcohol-based chemical vapours†

**DOI:** 10.1039/d2ra03931a

**Published:** 2022-08-03

**Authors:** Lerato L. Mokoloko, Joyce B. Matsoso, Nikolas Antonatos, Vlastimil Mazánek, Beatriz D. Moreno, Roy P. Forbes, Dean H. Barrett, Zdeněk Sofer, Neil J. Coville

**Affiliations:** The Molecular Sciences Institute, School of Chemistry. University of the Witwatersrand Johannesburg 2050 South Africa neil.coville@wits.ac.za; DSI-NRF Centre of Excellence in Catalysis (c*change), University of the Witwatersrand Johannesburg 2050 South Africa; Department of Inorganic Chemistry, University of Chemistry and Technology – Prague Technická 5, Dejvice 166 28 Praha 6 Czech Republic zdenek.sofer@vscht.cz; Canadian Light Source Inc. 44 Innovation Boulevard Saskatoon SK S7N 2V3 Canada

## Abstract

The application of N-doped carbon nanosheets, with and without embedded carbon dots, as active materials for the room temperature chemoresistive detection of methanol and/or ethanol is presented. The new carbons were made by converting 0D N-doped carbon dots (NCDs) to 2D nitrogen-doped carbon nanosheets by heat treatment (200–700 °C). The nanosheets exhibited a lateral size of ∼3 μm and a thickness of ∼12 nm at the highest annealing temperature. Both Raman and TEM analyses showed morphological transitions of the dots to the sheets, whilst XPS analysis revealed transformation of the N-bonding states with increasing temperature. PDF analysis confirmed the presence of defective carbon sheets. Room temperature screening of the chemical vapours of two alcohols (methanol and ethanol), revealed that the structure and the type of N-configuration influenced the detection of the chemical vapours. For instance, the lateral size of the nanosheets and the high charge density N-configurations promoted detection of both methanol and ethanol vapours at good sensitivity (−16.8 × 10^−5^ ppm^−1^_EtOH_ and 1.2 × 10^−5^ ppm^−1^_MeOH_) and low LoD (∼44 ppm_EtOH_ and ∼30.3 ppm_MeOH_) values. The study showed that the composite nature as well as the large basal area of the carbon nanosheets enabled generation of adequate defective sites that facilitated easy adsorption of the VOC analyte molecules, thereby eliminating the need to use conducting polymers or the formation of porous molecular frameworks for the alcohol detection.

## Abstract 2

2D layered carbon nanostructures made by annealing 0D carbon dots, have been used as ethanol/methanol sensors.

## Graphical abstract

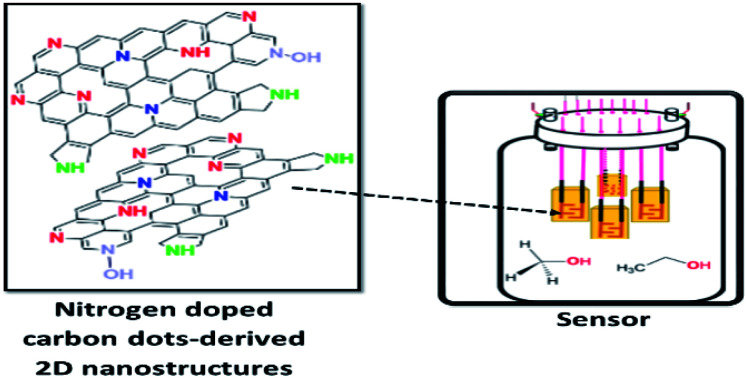

## Introduction

Volatile organic compounds (VOCs) are known to significantly reduce the quality of air and therefore cause harmful effects to humans and the environment.^1,2^ Unfortunately, a number of simple everyday activities such as painting, application of adhesives, use of cleaning products, production of cosmetics, and cooking, all lead to the emission of many VOCs.^3,4^ Additionally, alcohol-based VOCs such as methanol and ethanol are mostly found in alcoholic beverages, disinfectants and food products, and because of their volatility, impact our daily living.^3^ Therefore, the ability to detect and identify alcohol-based VOCs is required for the health and safety of humans, and the environment at large. Additionally, alcohol detection to ensure road safety protocols are obeyed, has also provided a large market for the detection of ethanol vapours.^5^ As such, the search for portable, low-cost, low power usage, high response and easily operated sensing devices for VOC detection still continues, in particular for those devices that can operate satisfactorily under room temperature conditions.^6^ Owing to this growing demand, several sensing techniques have been explored for the detection of VOCs. These include gas chromatography, infrared detection and chemoresistive detection.^7^ Among these various detection methods, chemoresistive devices are particularly useful, especially in terms of low power, easy operation, high selectivity and robust nature.^8^

Usually, the performance of the chemoresistive sensing devices is affected by the type of the active material used. Given this challenge, an arsenal of active materials, such as conducting polymers, semiconductors, metal oxides, chalcogenides and carbon allotropes, have been extensively studied.^8^ Amongst the various candidates, carbon allotropes, particularly graphene and its derivatives, are known to be promising active materials for the fabrication of room temperature chemoresistive sensing devices.^9,10^ Apart from using the graphene derivatives individually as active materials, various studies have shown that formation of composites of carbon allotropes with graphene derivatives can also significantly enhance their electrochemical properties.^11,12^ The synergy between the components of the composites has led to their enhanced sensing properties due to changes in the structural and electronic characteristics of the composites.^6,13^ For instance, Hu *et al.* reported that a composite of reduced graphene oxide and carbon dots (rGO–CDs) led to a highly selective and stable gas sensor under room temperature conditions.^14^ The enhanced sensitivity was attributed to the increased density of electron deficient sites as well as adsorption sites. Similarly, a composite of graphene, conductive polymer and carbon nanotubes on a glassy carbon electrode (GCE) was found to produce a methanol sensing device exhibiting fast response, high sensitivity, long-term stability and low-sample volume.^15^

Apart from the formation of carbon-based composites, tuning of the electronic properties of graphene derivatives without compromising the structural properties through intentional incorporation or doping with electron-rich nitrogen atoms provides an excellent route for increasing the materials binding sites. This is achieved by the creation of defective sites, while also inducing the formation of n- and/or p-type semiconducting properties, depending on the type of N-bonding configuration formed.^16–18^ A few reports have shown that these nitrogen-rich nanosheets, with targeted configuration, can be fabricated from facile synthesis procedures such as carbonizing metal–organic frameworks (MOFs) like indium-based coordinated polymers.^19^ Despite numerous reports on the synthesis of N-doped graphene derivatives, few studies have investigated the impact of the carbon and/or nitrogen-dopant domains on the sensing ability of N-doped carbon-based nanostructures.^20^ To explore this issue, and the effect of dopants, we have used CDs as carbon sources for the synthesis of large domain pristine or doped graphene derivatives.

The transformation of these 0D CDs to 2D graphene-like sheets occurs during the coalescence of CDs in the presence of a metal catalysts and/or upon exposure to temperature treatment.^21–23^ The transformation route takes advantage of the highly functionalized surface of CDs which subsequently enables easy incorporation of heteroatoms during the doping process.^24,25^ However, little is known on how N-doping impacts on this conversion process and the use of these doped materials as chemical vapour sensing materials. Therefore, to increase the scope of the CDs-derived graphene derivatives, the current study aims to explore N-doped graphene-like nanosheets derived from CDs as active materials for the detection of VOCs. The significant role played by the different nitrogen bonding states and the morphological changes associated with heat treatment on the room temperature sensing performance of these CDs-derived N-doped graphene-like nanosheets is thus described.

## Experimental

### Material synthesis and sensor preparation

The nitrogen doped carbon dots (NCDs) were synthesized using a modified microwave-assisted hydrothermal technique.^22,26^ Typically, 1.2 g of l-ascorbic acid (95%, Sigma Aldrich) and 1.2 g urea (CO(NH_2_)_2_, 99%, Sigma Aldrich) were stirred into 240 mL of deionised water, after which 60 mL of the homogeneous solution was transferred into four Teflon lined 100 mL microwave reaction tubes. The reaction tubes were then placed inside an Anton-Paar Multiwave 3000 SOLV microwave digester, equipped with the maximum power output of 1400 W and a controlled pressure of 60 bar. The reaction chamber was then subjected to microwave irradiation for 6 min at a power of 600 W, resulting in a temperature of 180 °C. After cooling the reaction chamber to room temperature, the product was obtained through centrifugation at 15 000 rpm for 15 min. The supernatants were filtered through a 0.22 μm poly(vinylidene fluoride) (PVDF) membrane (Membrane Solutions), followed by removal of water using a rotary evaporator, and drying at 60 °C. As previously described for the conversion of the undoped CDs,^22^ the effect of temperature on the NCDs was achieved by annealing the NCDs (500 mg) in a horizontal chemical vapour deposition (CVD) furnace for 10 min under 100 sccm of nitrogen (>99.999% N_2_, Afrox SA). After cooling to room temperature under N_2_ gas, the powdered samples were collected and labelled as NCDs200, NCDs400, and NCDs700 for the samples annealed at 200, 400, 700 °C, respectively.

Finally, for the electrochemical impedance spectroscopy (EIS) chemical vapour sensing measurements (Scheme S1†), the NCDs active materials were dispersed with sodium dodecyl sulphate (SDS, ≥98.5% Sigma Aldrich), as a surfactant, at a concentration of 0.5 mg mL^−1^. Thereafter, 10 μL of each solution was drop casted onto the ED-IDE 3-Au integrated electrode (Microflux Fluidic, Spain) and the electrodes were dried at 70 °C for 30 min. Determination of sensitivity to methanol (MeOH, ≥99.9%, Lachner) and ethanol (EtOH, ≥96%, Lachner) was achieved through addition of calculated concentrations (ppm, eqn (1)) of the analyte into the gas chamber:1
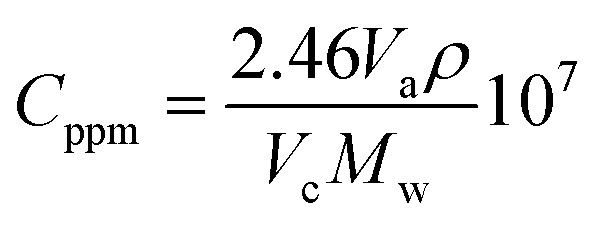
where *V*_a_ represents the volume (in μL) of the analyte added to the chamber, *V*_c_ is the volume (in mL, 50 mL) of the analysis chamber, *ρ* is the density of analyte (in gmL^−1^) and *M*_w_ is the molecular weight of analyte in g M^−1^. Responses based on EIS measurements were acquired in the 1–10^6^ Hz frequency range using the Metrohm Autolab potentiostat controlled by Nova 2.14 software after 5 min saturation. From an average of 3 fabricated electrodes, the response (Resp), limit of detection (LoD) and sensitivity (*S*) were determined by use of eqn (2)–(4):2
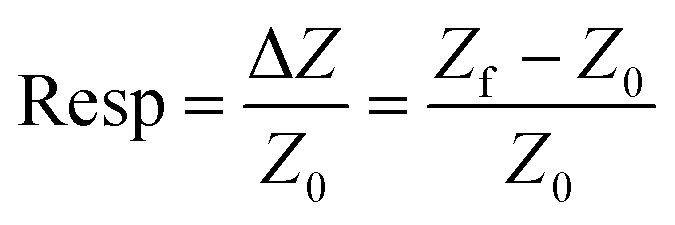

3 LoD = *Z*_b_ + 3*d*_b_

4
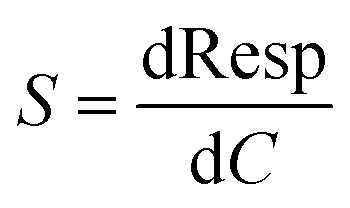
where, *Z*_f_ is the impedance of the sensor when exposed to analyte and *Z*_0_ the resistance of the sensor in ambient condition, *Z*_b_ is the average impedance, *d*_b_ is the standard deviation of the device impedance without analyte, and *C* is an analyte concentration.

### Characterization

The morphology of the active materials that have been immobilized on Si-O grids was acquired on an EFTEM Jeol 2200 FS microscope (Jeol, Japan), using 200 keV acceleration voltage, and HRTEM images were collected from a JEOL JEM 2100 fitted with a LaB6 gun. Their thicknesses were determined by atomic force microscopy (AFM) through use of a Ntegra Spectra microscope from NT-MDT. AFM measurements were performed in a tapping (semi-contact) mode under ambient condition with a scan rate of 1 Hz and scan line of 512 after drop-casting a sample suspension on a freshly cleaved mica substrate. ^13^C solid-state nuclear magnetic resonance (NMR) spectra were measured using a Bruker-500 MHz NMR spectrometer at a spinning frequency of 62.5 kHz (4.0 μs, 115 W). Raman spectra and solid-state photoluminescence (PL) spectroscopy data were collected using a Horiba LabRAM HR micro-Raman spectrometer (frequency double Lexel SHG argon ion laser). Raman measurements were done with a 514.5 nm laser and the PL measurements were done using a 244 nm excitation wavelength. Thermal stabilities of the samples were monitored by thermal gravimetric analysis (TGA) conjugated with weight loss derivative outputs (DTG) using a PerkinElmer 6000 thermogravimetric analyzer. Powder X-ray diffraction (PXRD) patterns were recorded on D2 phaser X-ray diffractometer using a Co Kα X-ray source operating at 30 kV and 10 mA. The measurements were taken between 2*θ* = 10–90°, with a 0.026° step. Total scattering data were collected on the Brockhouse high-energy wiggler beamline using a wavelength of *λ* = 0.2081 Å and a PerkinElmer XRD1621 area detector placed 160 mm after the sample. The data were processed using GSAS-II. The *Q*_max_ used to produce the PDF of the measured samples was 21 Å^−1^. The ESCAProbeP spectrometer (Omicron Nanotechnology Ltd, Germany) X-ray photoelectron spectrometer equipped with a monochromatic Al-α radiation source (1486.7 eV) was used for the wide-scan survey and high-resolution spectral measurements. The wide-scan surveys of all elements were measured in the range of 0–900 eV with a pass energy of 100 eV and a step of 1 eV, whilst high-resolution scans of the C1s, O1s, and N1s peaks were measured with a pass energy of 20 eV and a step of 0.1 eV.

## Results and discussion

### Temperature-dependent morphological evolution

**Fig. 1:**
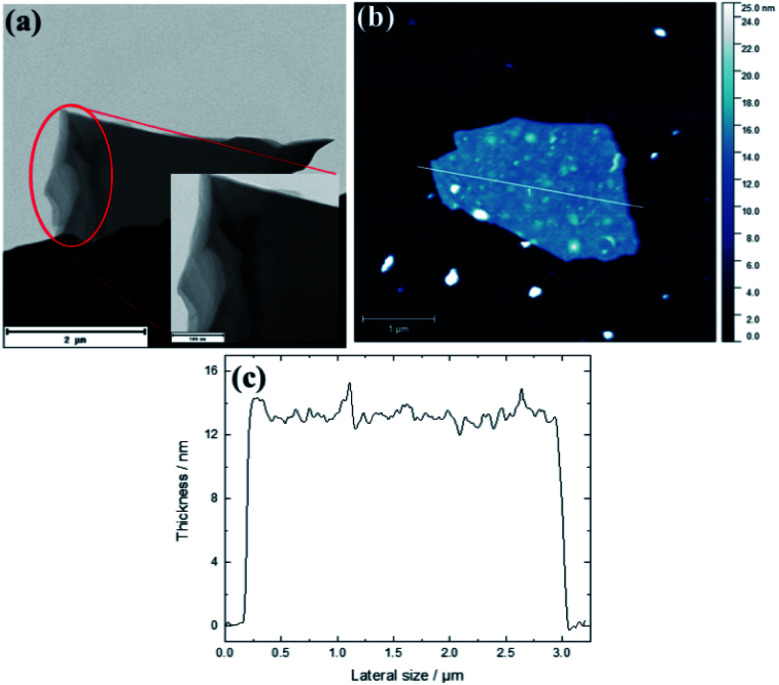
(a) Low magnification TEM, (b) AFM images and (c) height profile of the NCDs700 sample.

The morphological changes shown by the N-doped carbon dots (NCDs) as a function of heat treatment were investigated using both TEM and AFM micrographs. Both microscopic analyses showed changes in the lateral dimensions of the graphene-like nanosheets with heat treatment (Fig. 1, S1–S3†); from the small pristine NCDs of average particle size of ∼4.1 ± 0.9 nm (Fig. S1a†) to an amorphous mass (Fig. S1b†) and multi-layered nanostructures. For instance, TEM analysis showed that heat treatment at 700 °C produced large and multi-layered nanosheets with an average size of ∼3.71 ± 0.31 μm by ∼2.13 ± 0.91 μm (Fig. 1a). Additionally, the HR-TEM images of pristine NCDs and NCDs700 in Fig. S2† shows that the pristine NCDs had lattice fine structure, with the lattice *d*-spacing of *ca.* 0.204 and 0.210 nm, similar to that of the (101) and (100) plane lattice of graphite, respectively.^22,27^ In contrast, the NCDs700 did not show any lattice fringes (Fig. S2b†). This implies that graphitization is lost when the NCDs are transformed into 2D nanosheets.

AFM images of NCDs700 also confirmed the formation of large-area stacked nanosheets of up to 2.75 μm in lateral size and ∼14 nm in thickness (Fig. 1b and c). The formation of the nanosheets could be attributed to high temperature transformations of the carbon dots nanostructures, which was subsequently followed by the coalescence of the as-produced carbonaceous fragments into stacked two-dimensional (2D) nanostructures.^22,23^ In comparison to the undoped CDs-derived graphene-like nanosheets which exhibited an average size of *ca.* 1.71 × 0.909 μm,^22^ it can be concluded that N-doping affected both the temperature required to transform the CDs as well as the final structure obtained at the highest annealing temperature.

The 400 °C heat treatment was observed to not only lead to formation of the multi-layered 2D nanosheets (Fig. S1c†), but the stacked nanostructures also contained small accreted quasi-spherical nanoparticles (Fig. S3b†). This indicates that the 400 °C annealing temperature was insufficient to facilitate a complete transformation of the NCDs into active carbon fragments and consequently resulted to formation of the mixture of CDs and the 2D nanostructures. Height profile analysis by AFM showed the presence of small and thinner (∼0.75 nm) nanosheets exhibiting the lateral diameter of ∼0.15 μm (Fig. S3c,† red line). Further confirmation of the important effect of annealing temperature on the transformation of the NCDs was shown by annealing at 200 °C that resulted in formation of an amorphous-looking mass of carbon embedded with quasi-spherical nanoparticles (Fig. S1b and S3a†).

### Physicochemical properties

**Fig. 2:**
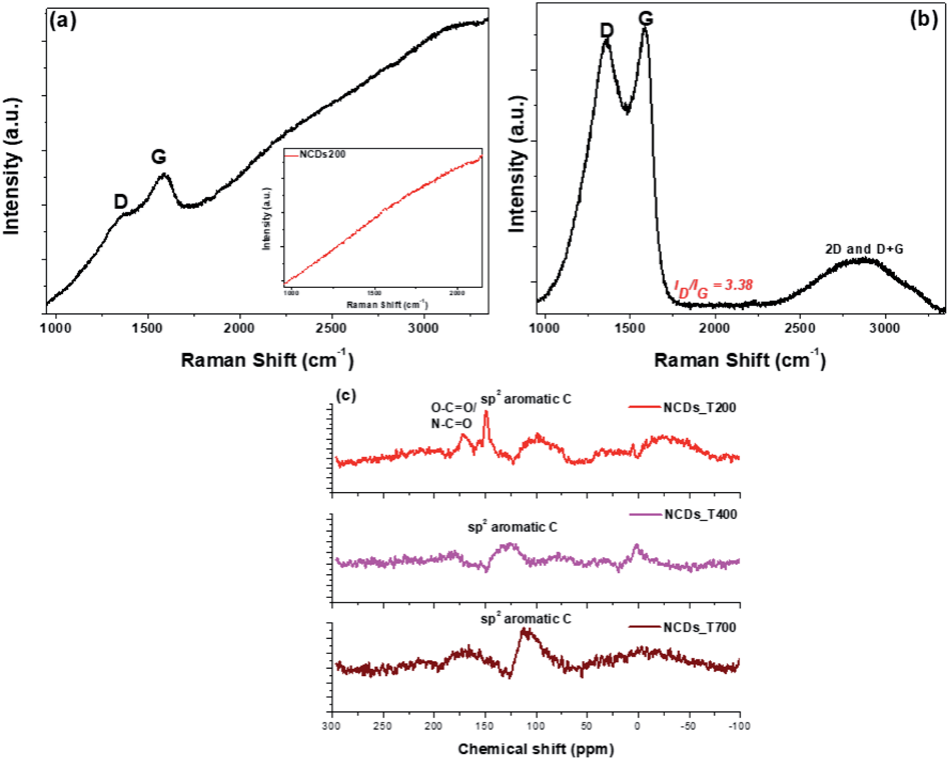
Raman spectra of (a) NCDs400 and (b) NCDs700 samples, with inset of (a) showing that of NCDs200; (c) ^13^C NMR spectra for the three annealed NCDs samples.

Structural disorder associated with the heat treatment and the presence of N-dopants were investigated using Raman spectra. As shown in Fig. 2, the spectra for the NCDs400 and NCDs700 samples exhibited fingerprint Raman peaks characteristic of carbonaceous materials.^28,29^ The first order Raman modes were located between ∼1341-1357 cm^−1^ and ∼1578–1580 cm^−1^ for the D-band and G-band, respectively, whilst the second order Raman (2D) band for the NCDs700, overlapped with the overtone of the D + G band at ∼2790 cm^−1^.^28,29^ In the case of the NCDs200 sample (Fig. 2 insert), the Raman modes could not be characterized due to the photoluminescent excitation of the carbon dots by the Raman radiation source as well as the fluorescence properties of the carbon dots.^30^ Additionally, the inclined baseline for the NCDs400 sample indicated that these materials still contained some fluorescent carbon dots. As such, quantification of the structural properties of NCDs400 sample was difficult. Nonetheless, quantification of the degree of graphitization for the NCDs700 sample was investigated from the estimated ratios of the integrated areas under the D-band and the G-band (*viz. I*_D_/*I*_G_).^28^ The structural defect density ratio (*I*_D_/*I*_G_) was estimated to be *ca.* 3.38 ± 0.12, resulting in a relatively large defect density (*n*_D_, see eqn (S2)†) of ∼11.6 × 10^11^ cm^−2^.^31^ Furthermore, the PXRD analysis results in Fig. S4a† show that increasing the annealing temperature did not yield highly ordered nanosheets. The low degree of graphitization for the NCDs700 sample was attributed to the realignment of the carbon lattice upon formation of larger graphitic domains as well as the lattice distortion associated with the incorporation of the nitrogen heteroatoms, confirmed by pair distribution function (PDF) analysis (see below). Therefore, taking into account the relation between the excitation wavelength and the defect density ratio, the average sp^2^ crystallite sizes (*L*_a_, eqn (S1)†^31^) was found to be ∼4.98 ± 0.03 nm. The *L*_a_ value complimented the TEM and AFM micrograph data (Fig. 1a and c), which revealed the formation of bigger lateral size nanosheets upon annealing at 700 °C, as was also observed from our previous work on undoped nanosheets. Interestingly, bigger sheets were formed for the N-doped samples.^22^

Based on the thermal stability results from TGA (Fig. S4b†), the first order derivative profiles of the NCDs400 and NCDs700 samples confirmed the formation of carbonaceous nanosheets as shown by the decomposition peak at ∼624 °C and 635 °C, respectively. The single peak indicated a homogenous decomposition of the highly defective 2D structured nanosheets and/or the carbon core.^32^ Furthermore, the peaks at *ca.* 343 °C and 380 °C for the NCDs200 and NCDs400 samples, respectively, suggested the removal of amorphous carbon phases and organic dangling-bonds from the NCDs upon heat treatment. The lack of the low temperature peak(s) for the NCDs700 sample is indicative of the formation of a CDs-free product.^22^ More importantly, the peak at *ca.* 500 °C for NCDs400 and the broadness of peaks for the NCDs700 samples can be linked to strongly bound functional groups.^33^ Taking into account the contribution of the N-configuration on the thermal stability of the NCDs samples, an increased thermal stability of ∼200 °C was observed for the NCDs samples as compared to their undoped counterparts.^22^ The observation may be attributed to the presence of the pyrrolic-, pyridinic- and graphitic-N bonds present in the nanosheets as suggested by the XPS results (see below). These groups are thus more stable under air, compared to the O-containing groups.^34^ Finally, the small derivative peaks between *ca.* 60 and 80 °C in all 3 samples were attributed to moisture found within the samples.^35^

^13^C NMR data were collected and found to be consistent with the TGA results, which showed removal of oxygen functional groups after high-temperature annealing (Fig. 2c). For instance, the ^13^C NMR spectra of the NCDs200 showed a peak at *ca. δ* 170 corresponding to O–C=O and/or N–C=O^36^ which decreased on annealing. More importantly, the sp^2^ peak at *ca. δ* 117 ppm increased with increasing annealing temperature which is characteristic of the presence of aromatic carbon atoms, hence signifying the transformation of the carbon into large-area nanostructures. Finally, PL spectra showed that only the NCDs200 was PL active, with an emission peak at *ca.* 609 nm, located in the near-IR region.^22^ This emission peak was due to the presence of the NCDs embedded within the formed layered nanostructures. The observed PL quenching that occurred after annealing (Fig. S4c†) is further suggestive of the removal of PL-active surface functional groups on the CDs,^37^ thereby leading to structural transformation from CDs to layered materials.

### Compositional analysis

**Fig. 3:**
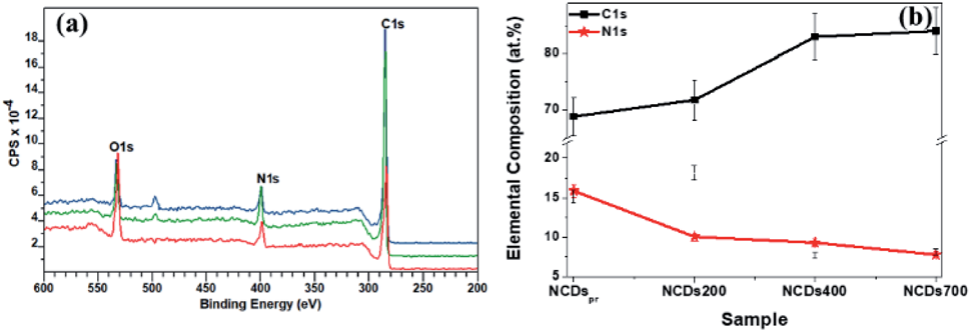
(a) XPS survey spectra for NCDs200 (red), NCDs400 (green), and NCDs700 (blue) samples, as well as (b) elemental composition of the as-synthesized samples.

Through the estimation of the integrated areas under the XPS wide scan spectra, the atomic percentage (at%) compositions of the annealed NCDs were determined (see Table S1†). From Fig. 3a, three distinct characteristic peaks corresponding to C1s (∼284 eV), N1s (∼398 eV) and O1s (∼532 eV) were observed. Detailed analysis of the survey spectra revealed the evolution of the nitrogen content with annealing temperature. For instance, the freshly prepared NCD sample showed the incorporation of *ca.* 15.9 at% of nitrogen heteroatoms, whilst treatment of 700 °C led to a decrease in the N content to *ca.* 7.79 at% within the carbon lattice (Fig. 3b). The decreasing N-content with increasing annealing temperature is consistent with the loss of edge-bonded nitrogen atoms in the form of nitrogen gas, ammonia gas and/or nitrogen radicals.^38^ Moreover, the high temperature promoted coalescence of the carbon domains, led to an increased carbon content on annealing (*ca.* 68.8 at% in the NCDs and *ca.* 84.1 at% in the NCDs700).

**Fig. 4:**
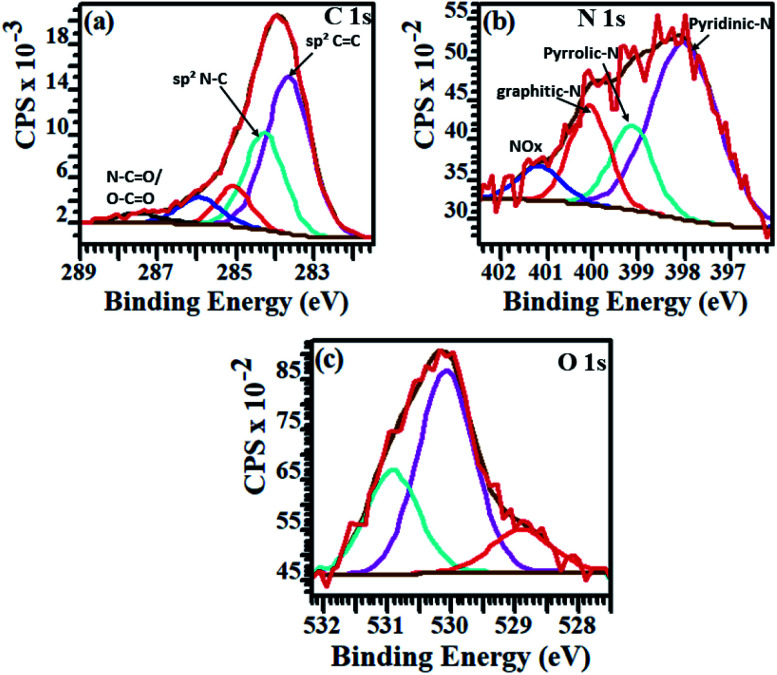
Peak fitted XPS curves for (a) C1s, (b) N1s, and (c) O1s of NCDs700 sample.

The bonding states of carbon (C), nitrogen (N) and oxygen (O) atoms in the annealed samples were determined through the peak fitting of their high resolution XPS spectra. Fig. 4 displays the resolved spectra for NCDs700 sample, whilst those of the NCDs200 and NCDs400 samples are shown in Fig. S5 and S6.† The broad, tailing C1s spectra (Fig. 4a, S5a and S6a†) was resolved to at least five components corresponding to graphite-like sp^2^ C–C bonds (∼283.6–283.8 eV), the sp^2^-N–C bonds (∼284.8–285.1 eV), the defect-induced sp^3^-C–C and/or C–N bonds (∼285.6–286.2 eV), and the oxygenated bonding configurations of C=O (∼286.6–287.7 eV) as well as the N–C=O and/or O–C=O bonds (∼287.9–288.8 eV).^39,40^

**Scheme 1:**
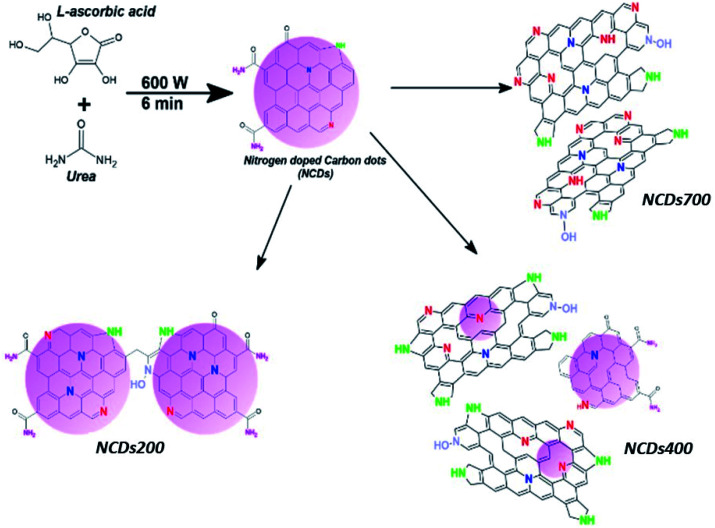
Representation of evolution of N-configurations in NCDs with respect to annealing temperature.

Similarly, the type of nitrogen dopant in the carbon lattice of the annealed NCDs samples was investigated by the deconvolution of the N1s spectra (Fig. 4b, S5b and S6b†). Peak fitting revealed four component peaks attributed to contributions from pyridinic-N (∼397.7–398.1 eV), pyrrolic-N (∼398.9–399.2 eV), substitutional/graphitic-N (∼400.1–400.3 eV), and the oxidized pyridinic-N bonds (NO_*x*_, ∼401.2–402.1 eV).^39^ Finally, the O1s spectra (Fig. 4c, S5c and S6c†) were deconvoluted into three component peaks which can be assigned to O=C and/or O–N (∼528.9–529.9 eV), O–C (∼530.1–531.6 eV), and the N–C=O/O–C=O (∼532.9–533.5 eV) bonds, respectively.^40^ Noticeably, the different bonding state distributions of the Nitrogen (Fig. S7†) showed the formation of different N-configurations through the adjustment of the annealing temperature (Scheme 1). For instance, annealing at 400 °C led to the formation of predominantly pyrrolic-N bonding states (∼45.8%) and pyridinic-N configurations (∼39.4%), thereby indicating that most of the amide groups from urea are bonded to the carbon at the domain edges. Moreover, fewer substitutional N-configurations (∼12.3%) show that the 400 °C heat treatment is not sufficient to facilitate the coalescence of the NCDs domains into graphene-like nanostructures. Such coalescence phenomenon could result in the transformation of pyridinic-N bonds into graphitic-N type.^38^ On the other hand, a higher annealing temperature (*e.g.* 700 °C) was found to enhance the formation of pyridinic-N (∼56.9%) due to the coalescence of the NCDs domains into bigger graphene-like nanostructures (Fig. 1a). The results are in good agreement with theoretical studies which have indicated that N atoms are more thermodynamically stable at carbonaceous edges; with the pyridinic-type being the most stable.^41,42^

### Total scattering measurements and derived pair distribution function (PDF)

**Fig. 5:**
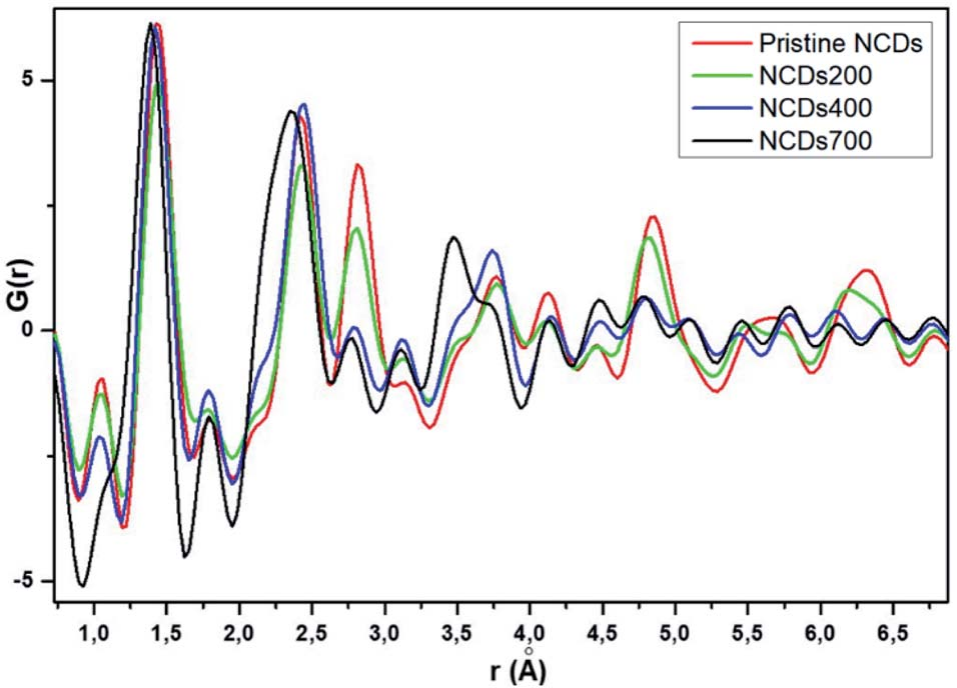
Zoomed region of the PDFs of pristine NCDs and annealed NCDs at 250, 400 and 700 °C.

Total scattering measurements were conducted at the Canadian Light Source to examine the local structure of the samples synthesized in this study (Fig. 5). The atomic pair distribution function (PDF) technique provides insight into the structure of materials in the short and medium range scales.^43^ Generally, the PDF results showed that the NCDs prior to annealing exhibited the longest range order, as can be seen by the increased dampening in the PDF signal from pristine NCDs to NCDs700 at *r* values as low as 10 Å (Fig. S8†). The first prominent peak, around 1.42 Å for the pristine NCDs (Fig. 5), corresponds to the C–C bonds and some C–N bonds with three nearest neighbours to carbons, all with sp^2^ bonding,^44^ bearing in mind that the pristine NCDs contains ∼15.9 at% N. In comparison with the C–C bonds at *ca.* 1.42 Å in a pristine graphene structure,^45^ the C–C peak is observed to shift to lower *r* values (∼1.38 Å) with N-doping and annealing, suggesting increasing contribution of the shorter C–N bonds. More importantly, the shifting and broadening of the peak could be ascribed to the varying contributions from both the pyridinic- and pyrrolic-N moieties, as these are characterized by peaks at *ca.* 1.34 Å and 1.38 Å, respectively.^44^

**Fig. 6:**
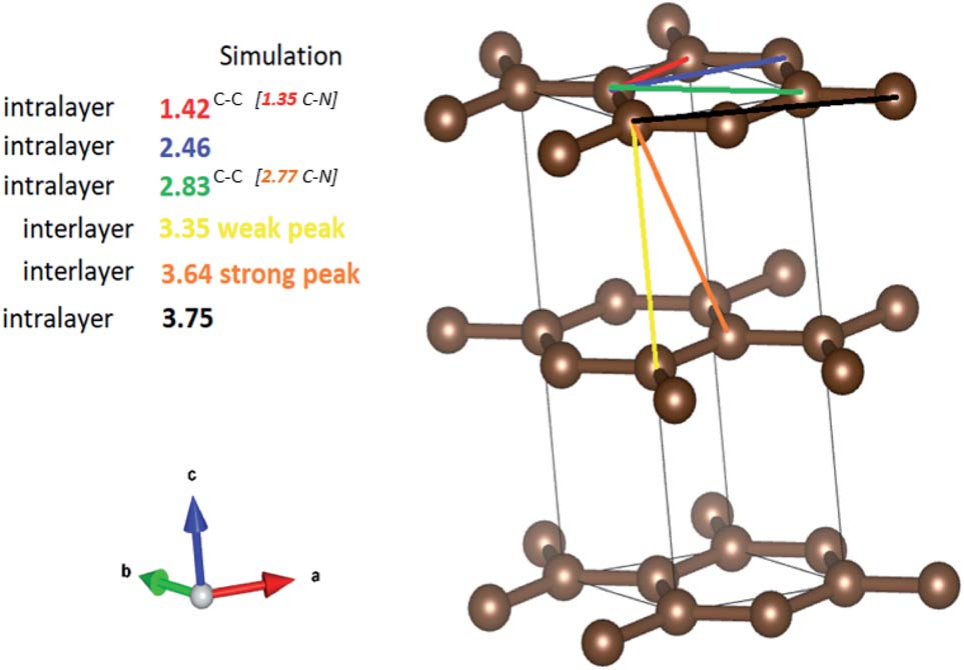
Inter and intralayer bond distances between carbon atoms in graphite. The values in brackets are given for nitrogen-doped graphene. These values correspond to the observed peaks in G(*r*).

The peak at 1.1 Å corresponds to C=O bond lengths with slight shortening of the bonds from their regular 1.2 Å length due to electron delocalisation over the graphene resonance structures.^45^ It must be noted that these peaks, even though weak, do demonstrate a clear trend showing a reduction in the peak intensity from the pristine NCDs to the NCDs700 sample indicating the removal of these groups as temperature increases. The second prominent peak at *ca.* 2.44 Å corresponds to the shortest diagonal in the carbon hexagon and is close to that of the predicted model (*ca.* 2.46 Å, Fig. 6). This peak broadened as the annealing temperature increased. Most noticeably, the NCDs700 sample showed significant broadening at this peak position compared to the other samples, indicating the distance between the three atoms coordinating the central carbon in the hexagon, or the shortest diagonal in the hexagon, varies significantly from one ring structure to the next. The third peak at 2.81 Å (close to the *ca.* 2.83 Å, Fig. 6), which is twice the first C–C distance, is the long diagonal in the hexagon.^46^ This peak shifts to lower *r* values (∼2.77 Å) and its intensity decreases with increasing annealing temperatures. The observed trends with higher annealing temperature suggest increasing disorder in the C-ring due to the insertion of N in the rings.

It is clear that the NCDs prior to annealing exhibit the longest range order, as can be seen by the increased dampening in the PDF signal from pristine NCDs to NCDs700. It is noted that the signal attenuation is high, even at *r* values as low as 10 Å, especially for NCDs400 and NCDs700, indicating the existence of short coherence lengths (Fig. S8†). Typically, the PDF pattern that originates from interlayer atomic correlations is signified by the weak 3.35 Å and strong 3.64 Å peaks (Fig. 6) and is indicative of well-ordered graphite structure. In all samples, the weak peak at 3.35 Å (Fig. 6) shifts to slightly higher *r* values (3.48 Å, Fig. 5) indicating a small increase in the interlayer spacing. A similar trend is noted when examining the 3.64 Å peak (Fig. 6) which is also shifted to slightly higher *r* values and is positioned around 3.75 Å (Fig. 5), with lower than expected peak intensity. Therefore, the shift of these peaks and the noted reduction in the intensity of the 3.75 Å peak indicates a higher degree of turbostratic and positional disorder from one graphene sheet to the next.^47^ Therefore, the aforementioned shifted peak positions to higher *r* values are due to disorder arising from the defective structures within the nitrogen-doped graphitic sheets leading to the slightly larger interlayer spacing coupled with turbostratic disorder.^47^ The data shows that by 700 °C, significant insertion of nitrogen into the structure has occurred, as well as the formation of higher-order ring structures and an increase in defective structures.

### Sensing properties

#### Chemoresistive behaviour of the sensors

**Fig. 7:**
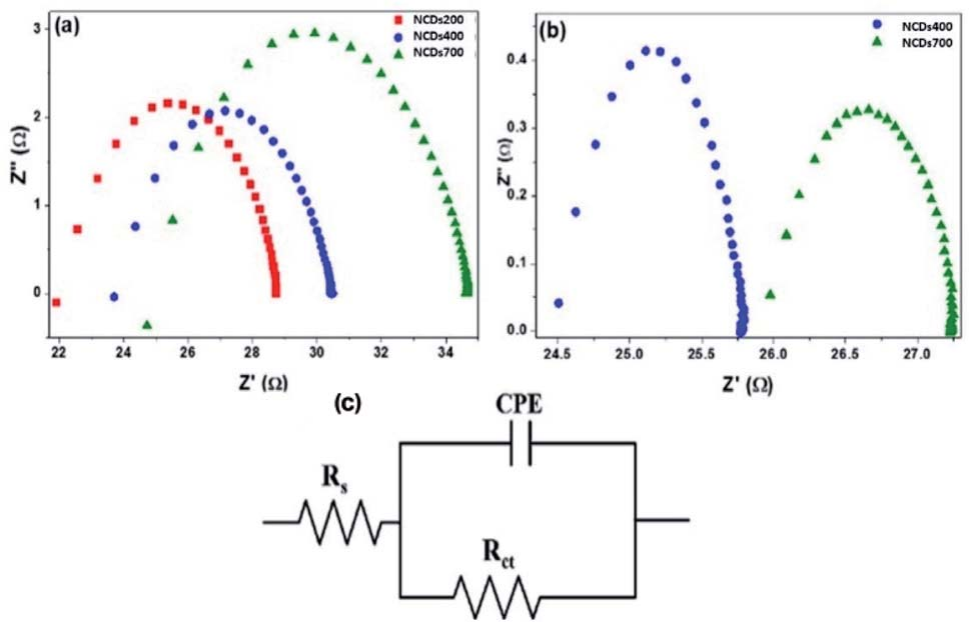
Nyquist plots for the sensor devices after exposure to (a) 1200 ppm of MeOH and (b) 1100 ppm of EtOH vapours, (c) representative equivalent Randle circuit.

Prior to the determination of the chemoresistive behaviour of the electrode materials, the sensors were screened for the detection of a range of VOCs (methanol, ethanol, acetone, isopropyl alcohol and acetonitrile). From this study, the NCDs400 and NCDs700 samples were found to be responsive to both methanol and ethanol, whilst NCDs200 sample was responsive to only methanol. As a result, detailed investigation of the chemoresistive behaviour of the active materials was performed by recording the Nyquist plots after exposure to 1200 ppm MeOH and 1100 pm EtOH (Fig. 7). For both analytes, the Nyquist plots exhibited a single semi-circle which indicated that, regardless of the analyte, the electrical interaction between the analyte and the electrode followed a parallel circuit connection with the impedance resistance parallel with the double-layer capacitance.^48^ More importantly, the relatively equal solution resistance (*R*_S_, Table 1) values for the detection of either MeOH or EtOH for the devices revealed similar charge transport properties of the accumulated analyte vapour solution-like electrolyte around the nanostructures.^49^

**Table 1:** Circuit parameters of the NCDs for detection of MeOH and EtOH

Analyte	Active material	Resistance (Ω)		Capacitance (μF)
		*R*_s_	*R*_CT_	
Methanol	NCDs200	22.5	6.11	0.18
	NCDs400	24.2	6.07	0.15
	NCDs700	25.6	8.95	0.10
Ethanol	NCDs400	24.3	1.47	17.9
	NCDs700	25.6	1.58	4.86

On the other hand, owing to the different structural properties and the varying composition of the N-dopants within the active materials, it was observed that each electrode responded differently to the two analytes. Specifically, the NCDs700 device exhibited the highest resistance in the presence of MeOH, followed by the NCDs200 and eventually the NCDs400 device, as shown by the values of the charge transfer resistance (*R*_CT_, Table 1). The faster charge transfer for the NCDs400 can be attributed to the abundance of pyrrolic-N (∼45.8%) and pyridinic-N configurations (∼39.4%) through an improved π–π interaction with the analyte molecules as well as the availability of extra π-electrons on the N-dopants for the ionization of adsorbed oxygen species.^50^ Moreover, the presence of a combination of NCDs fragments and small-sized graphene-like nanosheets could have created an abundance of defective sites which enabled quicker transport of charge-carriers. The slower charge transfer for the NCDs700 devices for detection of both MeOH (8.95 Ω) and EtOH (1.58 Ω) can be ascribed to the charging-hopping effect over the large lateral flakes,^50^ despite the material having electron-rich centres due to the presence of the graphitic-N (∼18.3%) and pyridinic-N (∼56.9%) configurations.^50^ Based on the results, it can be seen that not only the morphology of the carbon nanostructure, but also the concentration of the different nitrogen bonding states, plays a major role in the charge-carrier transfer properties of the NCDs.

#### Performance analysis

**Fig. 8:**
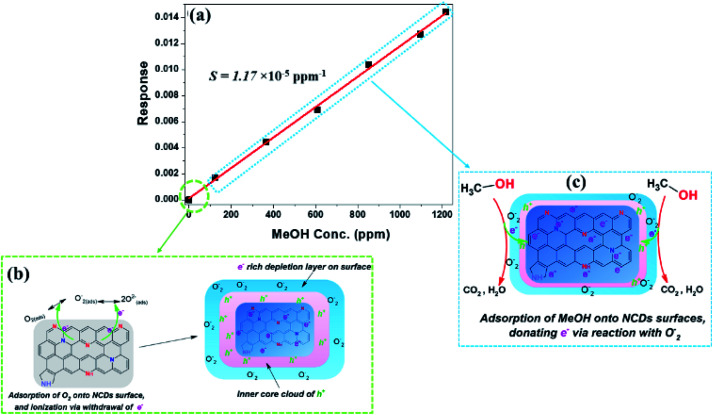
(a) Sensor response as a function of MeOH vapour concentrations for the NCDs700 sensor devices, (b) ionization of adsorbed O_2_, and (c) detection mechanism of MeOH.

**Fig. 9:**
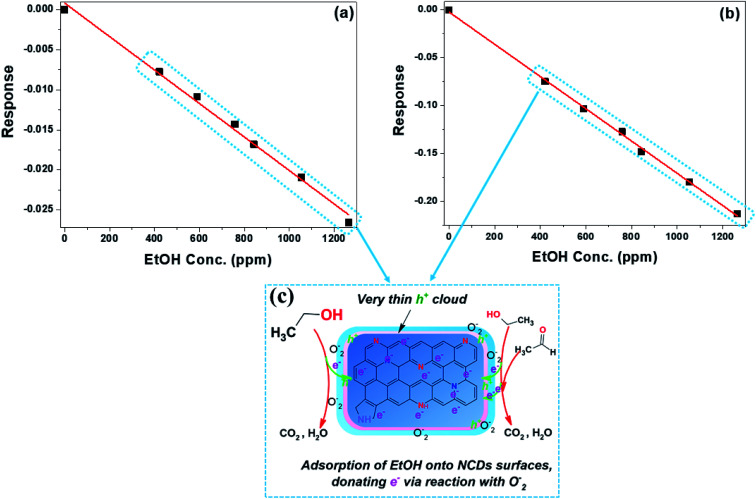
Sensor response as a function of EtOH vapour concentrations for the (a) NCDs400 and (b) NCDs700 sensor devices; (c) detection mechanism of EtOH.

Following the establishment of the significant roles played by the N-configurations on the electrochemical properties of the NCDs active materials, the sensing parameters of their corresponding devices for the detection of MeOH and/or EtOH vapours were then evaluated. The parameters were estimated through calculation of the concentration limit of detection (LoD, eqn (3)) and sensitivity (*S*, eqn (4)) from plots of sensor response (Δ*Z*/*Z*_0_, eqn (2)) against concentration (Fig. 8, 9 and S9†). Upon exposure to increasing concentrations of MeOH vapour, the sensor devices showed a p-type semiconducting behaviour^51^ as shown by the increasing sensor response with increasing analyte concentration (Fig. 8a and S9†). The observed p-type semiconducting nature, regardless of the presence of the n-type nitrogen dopants, is representative of the surface chemistry of the active materials. Normally, during the measurement of the sensor response and in the absence of MeOH (*i.e.* 0 ppm), ionization of the adsorbed oxygen molecules occurs, resulting in formation of O_2_^−^ or O^2−^ active species through withdrawal of electrons from the nanostructures.^52^ This resulted in the formation of an electron-rich depletion layer around the NCDs (Fig. 8b). Therefore, depending on the N-content as well as the structure of the NCDs samples, it can be anticipated that different thicknesses of the depletion layer will be formed around each active material. Consequently, the sensing parameters will then be influenced by the consumption of the depletion layer upon exposure to the analyte vapour. For instance, the extremely low sensitivity value for the NCDs700-based device (1.2 × 10^−5^ ppm^−1^, Table S2†) is indicative of a strong interaction of the MeOH molecules with the large-area electrode surface, thereby leading to compromised adsorption of MeOH and desorption of the by-products. On the contrary, the large-area, basal morphology of the nanosheets (*L*_a_ ≈ 4.98 nm) and the defective regions from both the N-dopants and carbon edges (*I*_D_/*I*_G_ ≈ 3.38) provided an abundance of adsorption sites for MeOH, as shown by the low LoD value (∼30.3 ± 0.7 ppm). Interestingly, an improved sensing performance for the detection of MeOH was observed for the NCDs200- and NCDs400-based devices (Fig. S9†), as evidenced by the large sensitivity values of 11.6 × 10^−5^ ± 0.6 ppm^−1^ and 4.36 × 10^−5^ ± 0.1 ppm^−1^, as well as low LoD values of 37.1 ± 0.3 ppm and 34.9 ± 0.5 ppm, respectively. The enhanced sensitivity could be attributed to the increased charge carrier density from an N-configuration such as the pyridinic-N (20.7–39.4 at%) and NO_*x*_ (2.5–9.9 at%) bonding states. Additionally, the NCDs/fragmented nanosheets composite nature of both NCDs200 and NCDs400 active materials created an abundant amount of charge capture sites, leading to a faster adsorption–desorption mechanism of MeOH molecules and their reduction products.

In the case of the sensing of EtOH, both the NCDs400 and NCDs700 sensor devices exhibited an n-type semiconducting behaviour^51^ as shown by the decreasing sensor response with increasing analyte concentration (Fig. 9). Lack of response to EtOH for the NCDs200 samples indicated that not only the type of N-configurations but also the structural properties of the active material influence the sensing mechanism for ethanol. For instance, the large sensitivity of −16.8 × 10^−5^ ± 0.5 pm^−1^ and low LoD value (43.5 ± 0.4 ppm) for the NCDs700-based sensor devices in comparison to the sensing performance of the NCDs400-based device (*S* = −2.09 × 10^−5^ ± 0.8 ppm^−1^ and LoD ≈ 91.4 ± 0.1 ppm) can be attributed to the weak interaction between EtOH molecules and the basal plane of the NCDs700. It is also possible that the slightly increased charge carrier density moieties in the form of graphitic-N (∼18.3 at%) and pyridinic-N (∼56.9 at%) bonding states play a role in the process. Therefore, the sensing mechanism for EtOH on the active materials can also be explained by the change in the surface depletion layer around the NCDs nanostructures (Fig. 9b and c). Similar to the sensing mechanism for MeOH, prior to exposure to the EtOH analyst vapour, an electron-rich depletion layer is formed on the surface of the NCDs (Fig. 8b) through ionization of adsorbed oxygen molecules. On exposure to the EtOH vapour, which acts as a reducing atmosphere,^53^ the ionized oxygen species are removed so as to convert EtOH into water and carbon dioxide. The removal of the ionized O_2_ species is then followed by the injection of electrons back into the NCDs nanostructures, thereby increasing the conductance channel within the NCDs structures (Fig. 9c).^54^

From Table 2, the reported impedimetric sensing performances for the detection of either MeOH or EtOH are seen to be comparable to data measured for graphene oxide, transition metal dichalcogenides (TMDs), metal oxides, and metal frameworks. More importantly, the facile route for transforming 0D CDs into 2D nanostructures^22^ also produced a composite of CDs/NCDs carbon fragments that improved the charge carrier retention and formation of the depletion layers. This facilitated a faster charge carrier transportation from the active material to the analyte molecules, and hence enhanced the sensitivity. Moreover, the use of the ‘composites’ (NCDs200 and NCDs400) as well as the NCDs700 (with large basal area) generated enough defective sites for improved adsorption of the VOCs analyte molecules, therefore eliminating the need to use conducting polymers^8^ or porous molecular frameworks.^55^ This provides an alternative method to make the alcohol sensor without added synthesis steps.

**Table 2:** Comparative MeOH and EtOH detection for EIS-based devices^a^

Analyte	Active material	Conc. range (ppm)	Temp. (°C)	LoD (ppm)	Ref.
Methanol	Au/TiO_2_-NT	200–600	27	—	56
	NCDs200	120–1200	RT	37.1	This work
	NCDs400			34.9	
	NCDs700			30.3	
	MoS_2_	200–400	200	—	57
	Cu-BTC MOF	250–1500	25	62	55
Ethanol	β-In_2_S_3_	500–1000	350	6^b^	58
	NCDs400	420–1200	RT	91.4	This work
	NCDs700			43.5	
	PLA-Gr	25–100	50	—	59
	GN-PMMA	1.96–69^c^	RT	19.7	8
	ZnO	1–500	RT	100	60

a Au/TiO_2_-NT/Ti → gold on titania nanotubes, In_2_S_3_ → indium selenide, ZnO → zinc oxide, PMMA → polymethyl methacrylate, MoS_2_ → molybdenum disulphide, Cu-BTC → copper-benzene tricarboxylate, PLA → poly(lactic acid), MOF → metal organic framework.

b Corresponds to response signal.

c Represents concentration in ppt.

## Conclusions

The study demonstrated that through a facile thermal treatment, nitrogen-doped and large area 2D graphene-like nanostructures could be easily attained from 0D CDs by adjustment of the CDs annealing temperature (200–700 °C). Furthermore, the nitrogen content as well as the nitrogen configurations of the N-doped CDs was found to be dependent on the structural transformation due to the annealing temperature. For instance, as higher temperatures favoured the lateral growth, the arrangement of N-atoms was found to be more dominant at the carbon edges (∼57 at% pyridinic-N), whilst the coalescence of the CDs into 2D nanostructures led to the ingrowth of interstitial nitrogen dopants (∼17 at% graphitic-N). At 200 °C the transformation of CDs into 2D nanostructures was incomplete, resulting in the synthesis of a carbon composite made of CDs fragments embedded in an amorphous mass of N-doped carbon. Screening for VOCs through the impedimetric sensing measurements, demonstrated that the NCDs-based devices were responsive to methanol and ethanol. A good correlation between the structural properties of the NCDs as well as the type of N-configurations and their corresponding sensitivity for the alcohol-based VOCs was observed. In particular, the large-area 2D NCDs700 nanosheets facilitated a high charge density *via* incorporation of pyridinic-N (∼57 at%) and graphitic-N (∼18.3 at%) configurations. This subsequently promoted enhanced sensitivity and selectivity of the NCDs700-based devices for the detection and recognition of both methanol and ethanol. Generally, the transformed nanostructures exhibited excellent room temperature impedimetric sensing performance for the detection of alcohol-based VOCs, thereby opening up a new platform for carbon-based materials for chemical vapour sensing. As the different NCDS gave different abilities for the detection of EtOH and MeOH this suggests that a combination of these ‘easy to make’ structures could be used to develop an ‘*electronic nose*’ for the room temperature detection of many other VOCs.

## Author contributions

L. L. M.; J. B. M: writing, data collection, analysis, design.

N. A., B. D. M.; D. H. B.: data collection, analysis.

R. P. F.; Z. S.; N. J. C.: conception, design, supervision, funding.

## Conflicts of interest

There are no conflicts to declare.

## Supplementary Material

RA-012-D2RA03931A-s001
