# Supplementary material for: From 0D to 2D: N-doped carbon nanosheets for detection of alcohol-based chemical vapours
Source: RSC Adv. 2022 Aug 3;12(33):21440–51. doi: 10.1039/d2ra03931a (PMC9346501; doi:10.1039/d2ra03931a)
Supplement: RA-012-D2RA03931A-s001 [file RA-012-D2RA03931A-s001.pdf]

Electronic Supplementary Material (ESI) for RSC Advances.
This journal is © The Royal Society of Chemistry 2022

Supplementary Information

From 0D to 2D: N-doped graphitic nanosheets for detection of alcohol-based chemical vapours

Lerato L. Mokoloko[a,b], Joyce B. Matsoso[c], Nikolas Antonatos[c], Vlastimil Mazánek[c], Beatriz D. Moreno[d], Roy P. Forbes[a,b], Dean H. Barrett[a,e], Zdeněk Sofer[c]*,Neil J. Coville[a,b]*

[a]The Molecular Sciences Institute, School of Chemistry. University of the Witwatersrand, Johannesburg 2050, South Africa

[b]DSI-NRF Centre of Excellence in Catalysis (*c**change), University of the Witwatersrand, Johannesburg 2050, South Africa

[c]Department of Inorganic Chemistry, University of Chemistry and Technology -Prague, Technická 5, Dejvice, 166 28, Praha 6, Czech Republic

[d]Canadian Light Source Inc., 44 Innovation Boulevard, Saskatoon, SK S7N 2V3, Canada

[e]National Research Centre for Energy and Materials (CNPEM), Rua Giuseppe Máximo Scolfaro, 10,000 - Polo II of High Technology of Campinas - CEP 13083-100, Campinas, SP, Brazil

*Corresponding authors: zdenek.sofer@vscht.cz; neil.coville@wits.ac.za

Results:

Calculation of the average sp² crystallite sizes and defect density were based on equations S1 and S2

$$L_a(nm) = 2.4 \times 10^{-10} \times \lambda_{laser}^4 \times I_G/I_D \quad \text{(S1)}$$

$$n_D(nm) = \frac{2.4 \times 10^{22}}{\lambda_{laser}^4} \times I_D/I_G \quad \text{(S2)}$$

where, λ_{laser} is the wavelength of the excitation laser of the instrument, in this case being 514.5 nm and I_G/I_D is the ration of the integrated areas beneath the G and D bands.

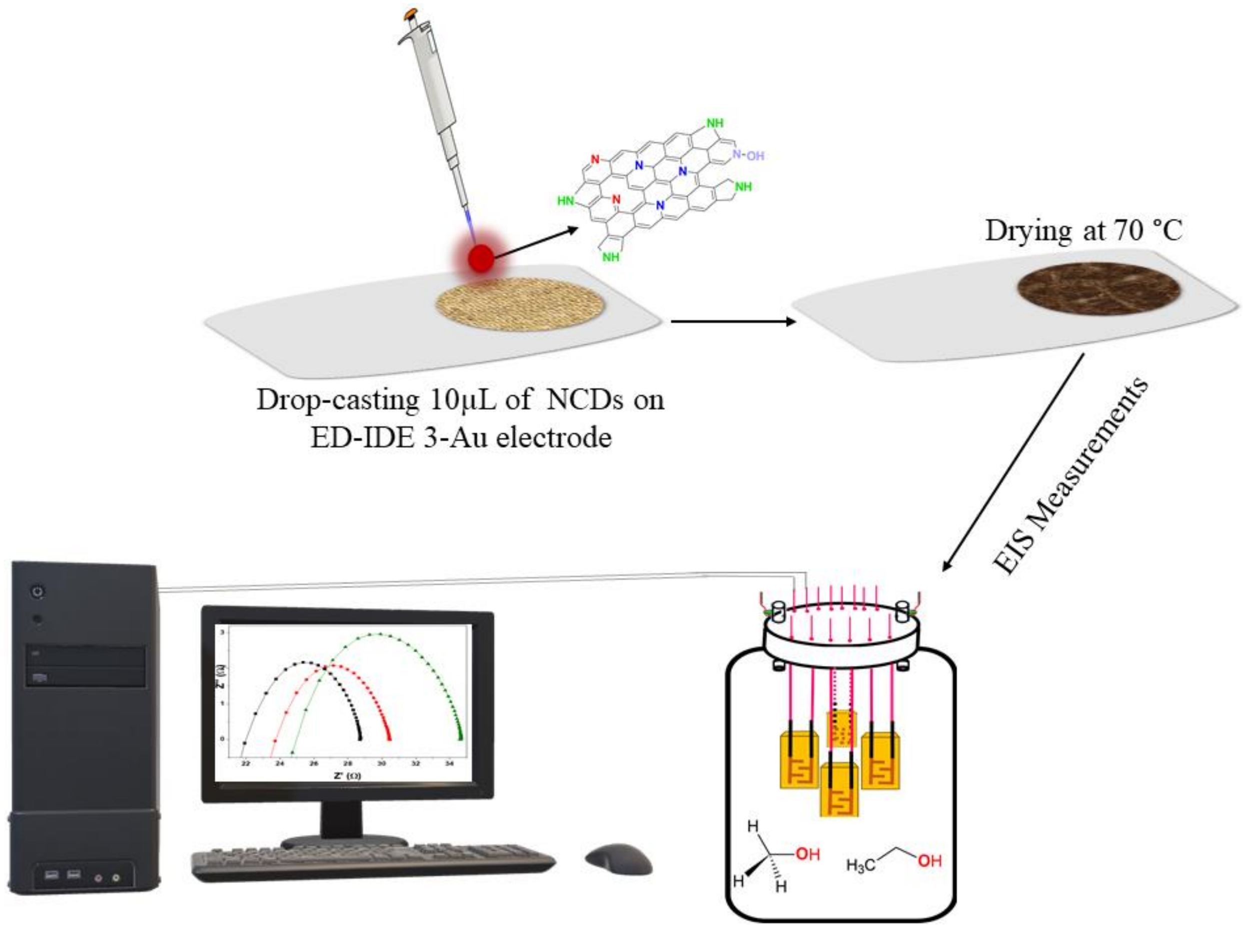

Scheme S1: Sensing device preparation and sensing set-up.

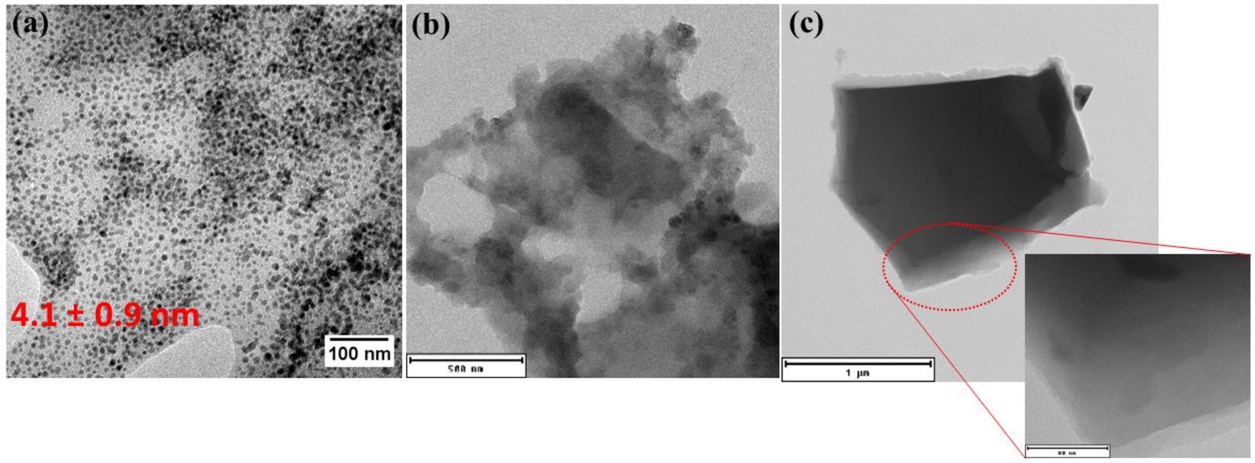

Figure S1: Low magnification TEM micrographs of (a) pristine NCDs, (b) NCDs200, and (c) NCDs400 samples.

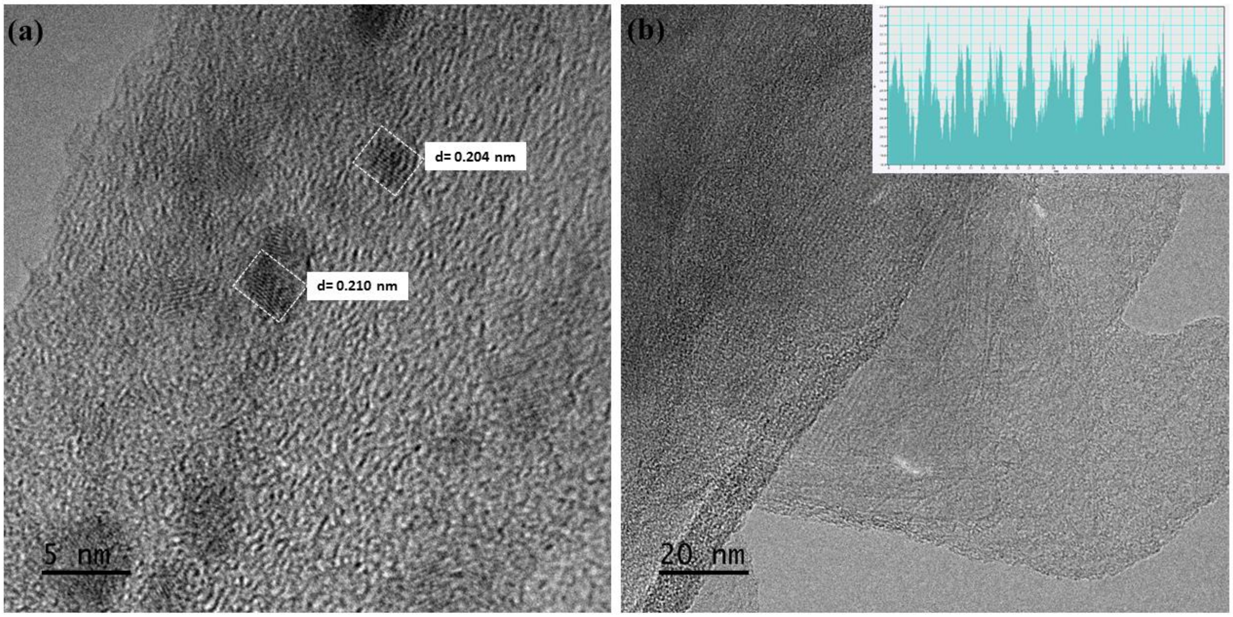

Figure S2: High resolution TEM micrographs of (a) pristine NCDs and (b) NCDs700 samples, with an inserted line profile. This does not show lines of similar heights or spacing between the lines indicating that theNCDs700 sample does not have graphitic character.

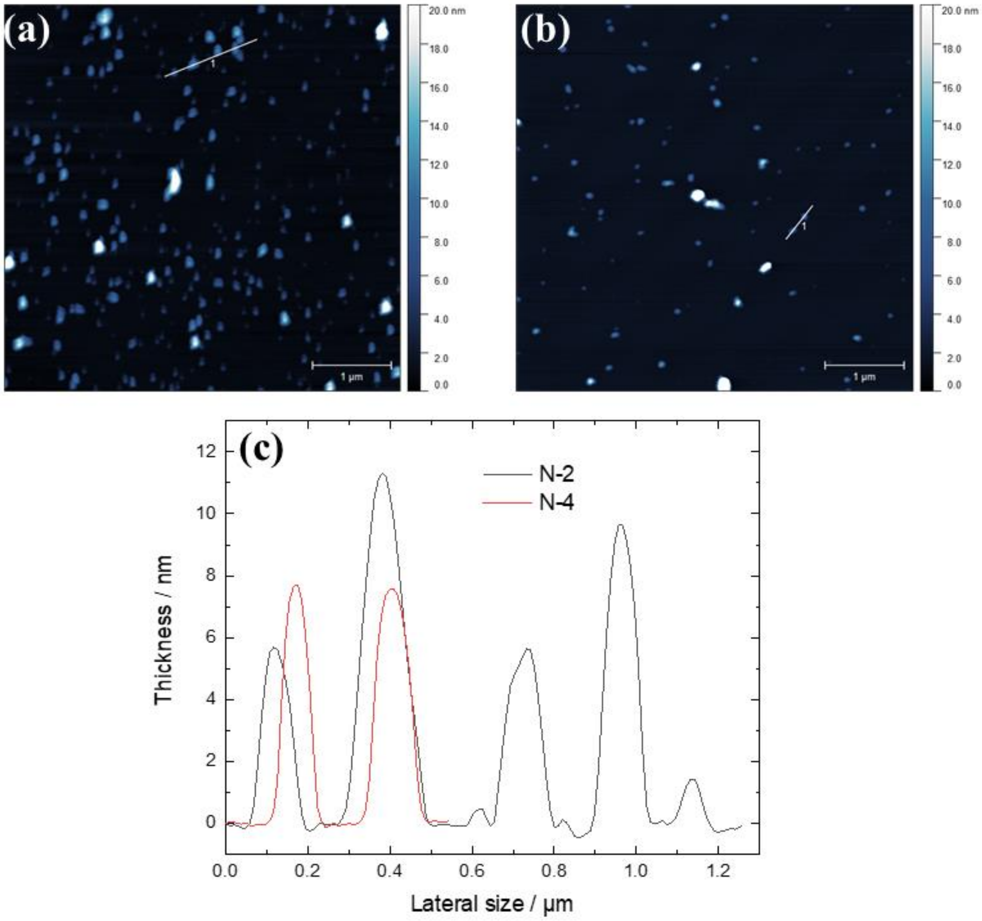

Figure S3: AFM images of (a) NCDs200 and (b) NCDs400 samples as well as (c) their corresponding height profile.

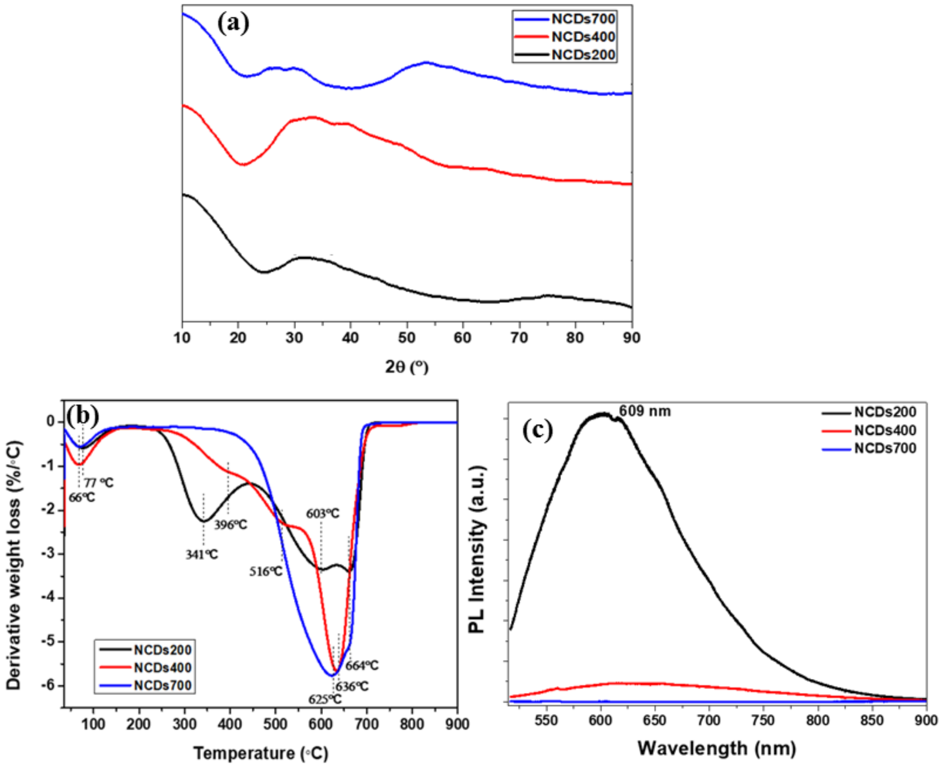

Figure S4: (a) PXRD patterns, (b) DTG profile and (c) PL spectra of the N-doped samples after annealing from 200 °C to 700 °C.

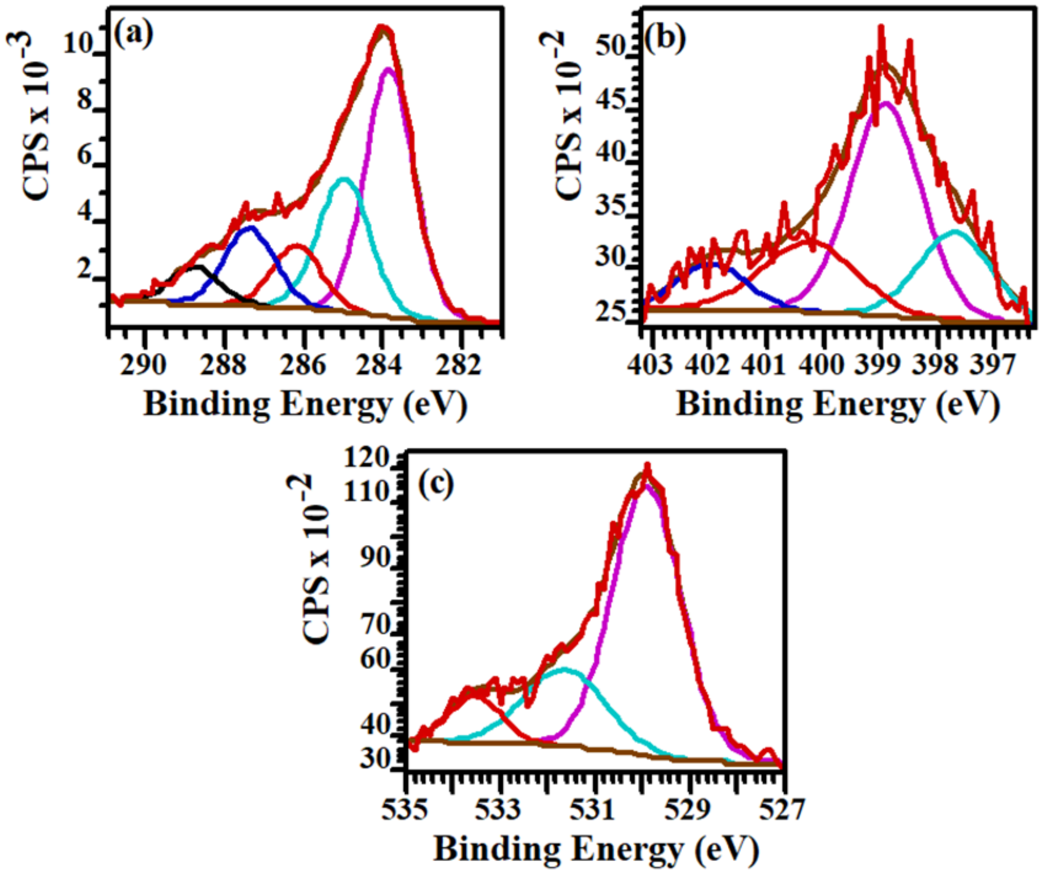

Figure S5: Deconvoluted (a) C1s, (b) N1s, and O1s spectra of NCDs200 sample.

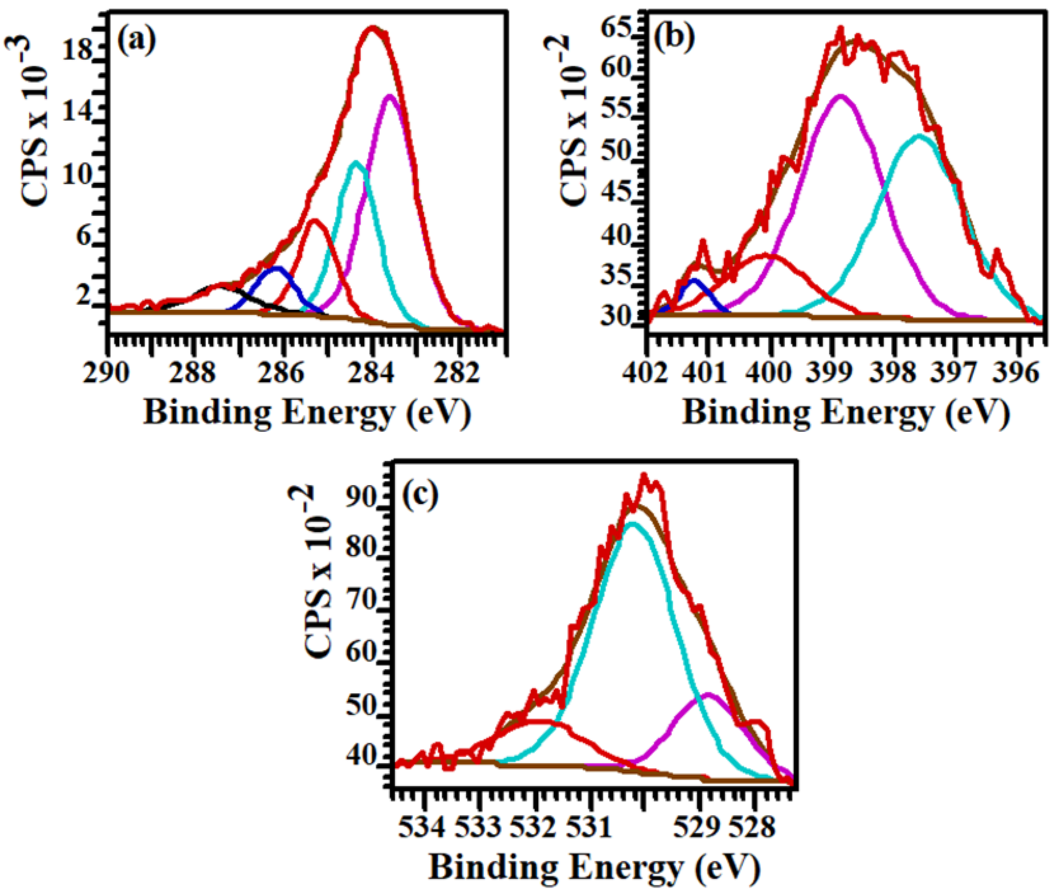

Figure S6: Deconvoluted (a) C1s, (b) N1s, and O1s spectra of NCDs400 sample.

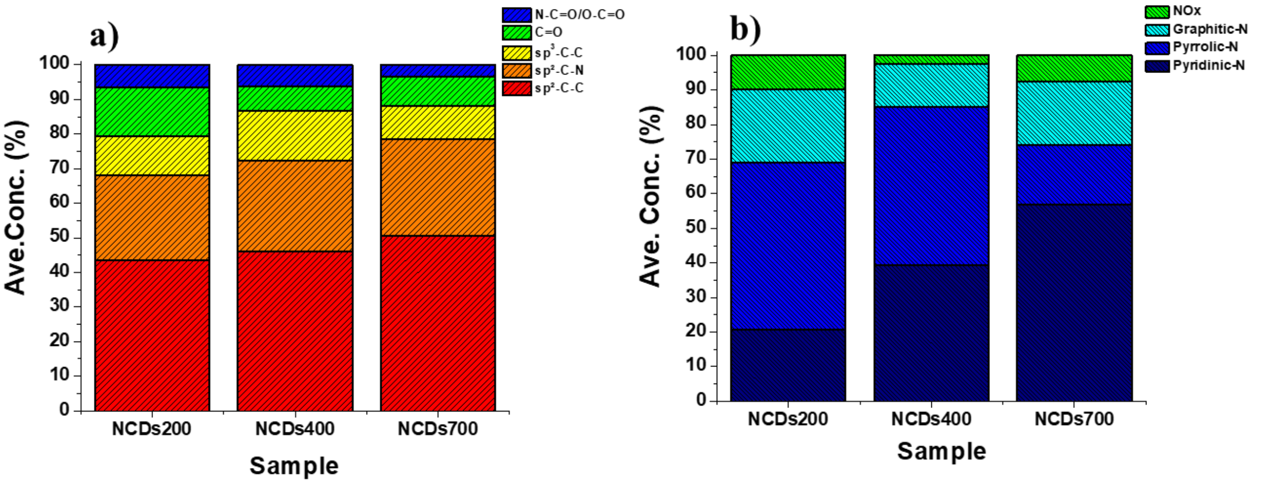

Figure S7: (a) Carbon and (b) Nitrogen bonding state distributions for all samples.

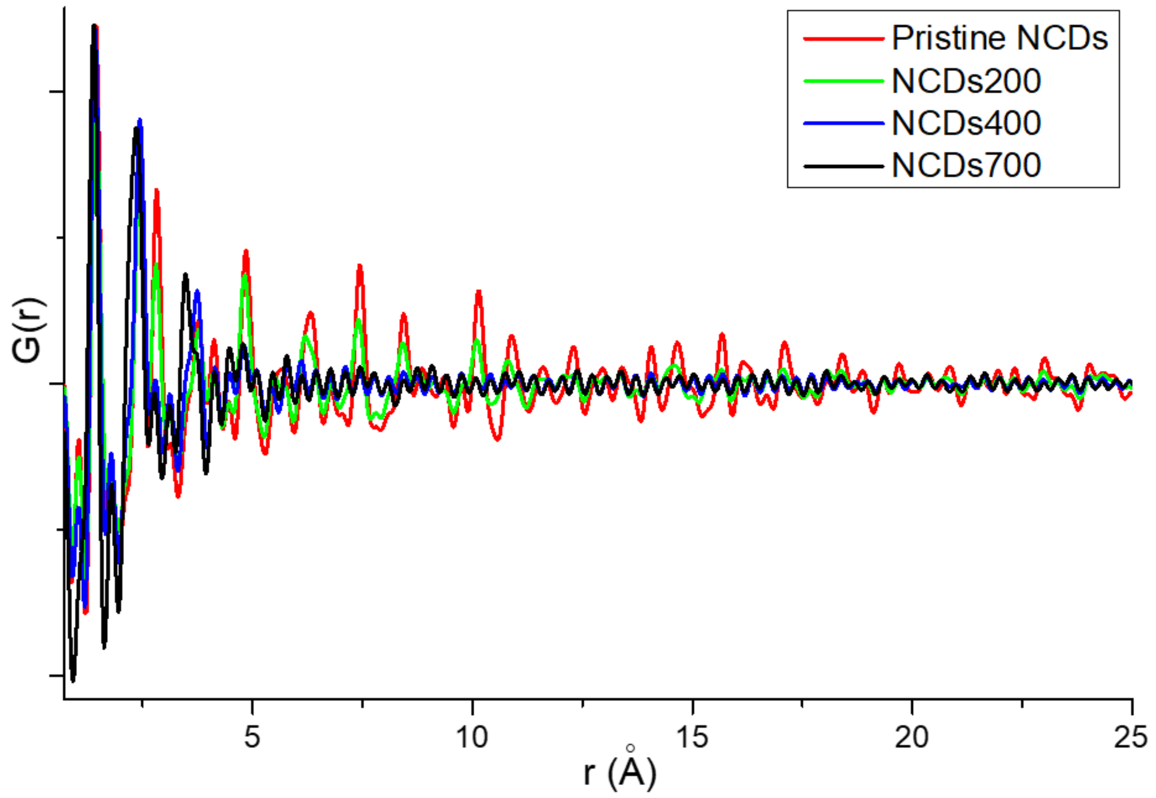

Figure S8: PDFs of pristine NCDs and annealed NCDs200, NCDs400 and NCDs700.

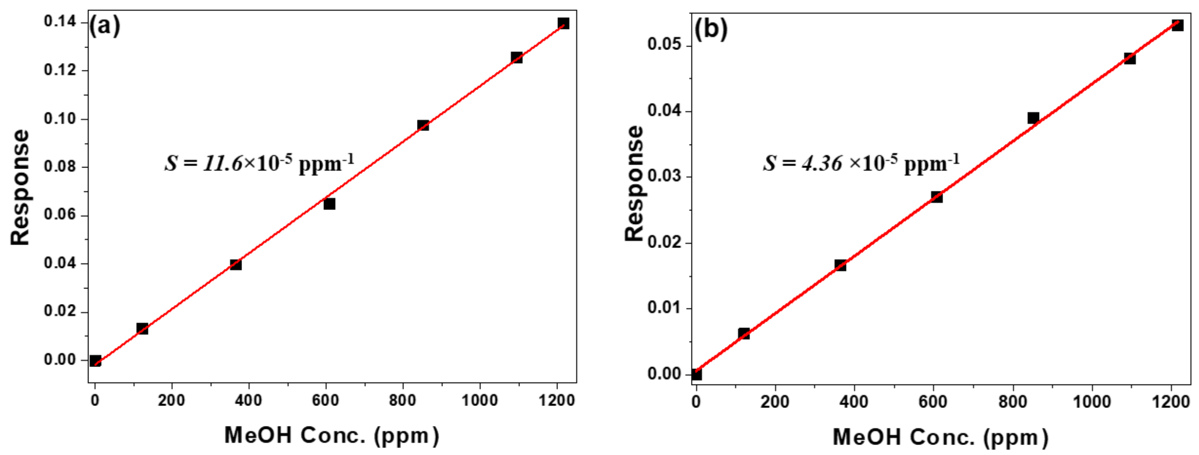

Figure S9: Sensor response as a function of MeOH vapour concentrations for the (a) NCDs200 and (b) NCDs400 sensor devices.

Table S1: Atomic compositions of the NCDs samples

Samples	Elemental Composition (at.%)		
	**C1s**	**N1s**	**O1s**
**NCDs200**	71.76	10.07	18.17
**NCDs400**	83.00	9.32	7.68
**NCDs700**	84.05	7.79	8.16

Table S2: Sensing parameters of the NCDs devices for detection of MeOH and EtOH; sensitivity (*S*, ppm^{-1}) and LoD (ppm)

Analyte	Electrode	*S* ($\times 10^{-5}$ ppm^{-1})	LoD (ppm)
**Methanol**	**NCDs200**	11.6 ± 0.6	37.1 ± 0.3
	**NCDs400**	4.36 ± 0.1	34.9 ± 0.5
	**NCDs700**	1.17 ± 0.2	30.3 ± 0.7
**Ethanol**	**NCDs400**	-2.09 ± 0.8	91.4 ± 0.1
	**NCDs700**	-16.4 ± 0.5	43.5 ± 0.4
